# Participatory design based on opinion pooling

**DOI:** 10.1098/rsta.2024.0108

**Published:** 2024-11-13

**Authors:** Michael Batty, Tianqu Shao, Fulvio D. Lopane

**Affiliations:** ^1^ Centre for Advanced Spatial Analysis, University College London, 90 Tottenham Court Road, London W1T 4TJ, UK

**Keywords:** participatory geodesign, land suitability mapping, overlay analysis, hierarchical weighting, Markov averaging, opinion pooling

## Abstract

We focus here on methods for locating future urban development, ranging from entire towns to site designs. These methods articulate the urban design problem in terms of a series of factors pertaining to different measures of land suitability that are represented as spatial surfaces or maps. In realistic problems, these factors inevitably conflict with one another, and we thus define various design methods that enable us to select optimal locations for development based on weighting these factors in different ways. We begin with methods for resolving conflicts between the suitability maps using simple averaging with equal weights and then introduce methods for representing the interactions between the factors as a hierarchy for how these factors can be related to each other following an order for their differential weighting. We then generalize this method to ways in which a variety of individual experts can pool their knowledge of the problem to achieve a consensus solution using a process of group dynamics. The implication is that these kinds of dynamics might be used to enable effective public participation where conflicts between opinions can be resolved collectively. We are currently exploring this through methods of geodesign where networks of stakeholders, planners and designers can be brought together to solve these problems both empirically as well as formally.

This article is part of the theme issue ‘Co-creating the future: participatory cities and digital governance’.

## 1. Science in design and city planning

Until the middle of the last century, those who sought to improve the quality of life in cities largely assumed that such systems should be designed and managed from the top-down. This was predicated on the basis that this approach would increase their efficiency, improve their equity and enhance their visual beauty. The dominant tools that were used to generate better cities—‘ideal’ or ‘optimal’ cities as they are called—were largely based on the visual and organizational intuition of the architects and planners involved and it was widely assumed that the sort of complex order that cities exhibited could be handled by professional designers whose skill was largely acquired in practice. This paradigm began to change as it was slowly realized just how complex city systems might be and a new science began to be fashioned. Jane Jacobs [1] was in the vanguard of such a movement, but it has taken more than half a century for it to come to fruition.

The process of design however has largely remained beyond the rudiments of this science, and although considerable advances have been made in the science of cities much of it originating from complexity theory [2], the process of designing the ideal city has largely remained resistant to formal explorations. In the last century, the notion that optimal cities might be derived from the top-down or even evolved from the bottom-up never really took off and only now are there glimpses of formalized ways in which urban design might be articulated. Generative grammars are now in the vanguard of such developments, but a science of design is yet to fully emerge [3,4]. However, in this article, we will sketch the rudiments of such a science where we begin with ideas associated with individual designers, experts working largely in isolation from one another, and then suggest how we can construct networks linking together designers in a participatory manner to synthesize their intuitions in deriving ideas about optimal cities. We will develop a series of structural models of the design process based on networks that link actors who focus their individual intuitions on design solutions. In short, we will present methods for modelling the design process from individual forms of action to group dynamics that are based on methods for pooling ideas about optimal cities held by different stakeholders.

We begin by sketching the origins of individual expert-orientated design, which is largely the prerogative of individual designers although single individuals rarely act in isolation, notwithstanding the association of a design idea with a particular person or group. Even prior to the development of design based on group dynamics, plans for optimal cities—generically referred to as master plans—were regarded as too complex for a single individual to design. Bringing together all the data and the intuition required for producing such a design was always a mammoth task [5]. If you examine how the greats such as Le Corbusier and Frank Lloyd Wright evolved their plans for the optimal city, they gathered together groups of architects, often having the role of ‘apprentices’ who would help them in pursuing such tasks. To illustrate these ideas, we will begin by outlining a relatively simple design problem that involves the search across a landscape for the best location for a development, such as a large housing estate or a new town. This is only one of many kinds of design problem, but it can be clearly articulated as one in which a series of relevant factors can be defined in such a way that they are comparable and commensurate in ways that they can be combined to produce good designs for best locations. These factors can be defined as spatial layers of different land suitability for housing, for example, or any relevant land development and the basic individual design problem is to combine these layers into a composite surface that defines the most suitable land.

We will then examine the problem of weighting these layers in different ways, drawing on notions that the layers can be clustered into different subproblems and then illustrating how these subproblems define a hierarchy that shows the order in which the layers can be combined. This individual problem can then be generalized to embrace different design viewpoints—different stakeholders—who hold different views about the degrees of land suitability for defining the optimal solution, the optimal plan. We will use a process of ‘opinion pooling’ to achieve convergence, which is a consensus between these different viewpoints and this establishes a method for defining a weighted suitability layer from which the best plan is derived. To indicate how this might be improved further, we briefly conclude with hints at how these processes resemble machine learning based on forward and back-propagation. The implications are that these methods might be used in future developments to determine the weightings and the ultimate suitability of the final layer.

## 2. The origins of design methods in map overlays

**Figure 1.**
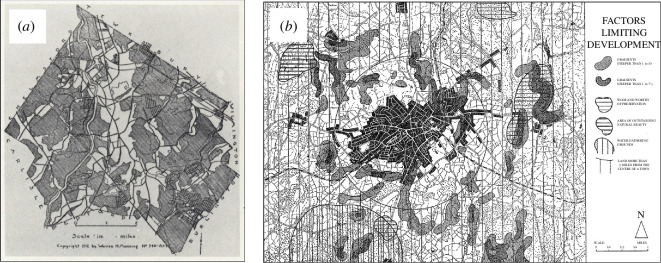
(*a*) Manning’s 1912 Plan based oncomposite suitability, see note 6. (*b*) Keeble’s [7] map overlays defining land suitable for development (with white most suitable in both examples; adapted from [6,7]).

Locating different land uses or activities within any kind of landscape can be conceived of as a process of identifying different levels of land suitability and then choosing the most suitable or a composite for locating the land use in question. The process of making comparisons between different degrees of land suitability goes back to the design of urban parks in the late nineteenth century with Frederick Law Olmsted suggesting that designers should identify different zones of suitability. In fact, it was Manning, an associate of Olmstead, who first demonstrated the method of identifying different factors with different levels of suitability for development across a landscape and then combining the layers using a process of map overlay analysis. This could thus be seen as a process for filtering or sieving out the most suitable areas [6]. We show Manning’s method in figure 1*a* where different layers are combined for the town of Billerica near Boston associated with a plan designed in 1912.

The method is intuitively obvious and was widely used in the first half of the last century to identify the most suitable areas for urban development during a time of rapid growth and suburban sprawl in more urbanized industrialized countries. The overlay method was widely exploited in early textbooks on planning such as that by Keeble [7], who showed how the methods could be adapted to many different kinds of location problem, particularly the design of new highways, new towns and urban development in general. We show a typical overlay map from Keeble’s text in figure 1*b*, and a good summary of the process is presented by Jaqueline Tyrwhitt [8] in her chapter in the APRR *Town and Country Planning Textbook* published in 1950.

**Figure 2.**
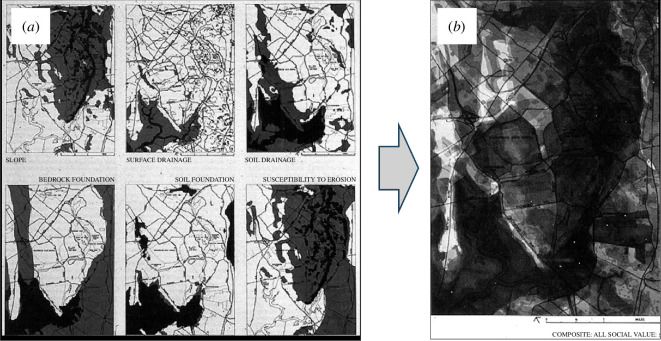
(*a*) Six physiographic factors that when equally weighted yield (*b*) the suitability surface (where white is most suitable for location of a highway) (adapted from [9]).

One of the most accessible examples of overlay analysis was presented by McHarg [9] in his book *Design with Nature*. There he demonstrated the best location for a highway as a consequence of a series of map overlays defining how different factors implying different levels of suitability could be combined in simple additive terms to locate the best corridor for the highway. Again, McHarg implied that the method was generic, hence applicable to different types of location problem. We show some factors from the typical problem in his book in figure 2 where we combine six physiographic factors—slope, surface drainage, soils drainage, bedrock foundation, soil foundation and susceptibility to erosion—using an equal weighting for each layer that yields the overall suitability map on the right of the figure.

There are several ways of augmenting McHarg’s process and a series of extensions and modifications of his methods have more recently been elaborated by Steiner *et al*. [10]. These suggest that in using such models to identify the most suitable locations, other factors need to be included to modify the combined suitability scores with respect to qualitative factors. The process of weighting each layer begs the question as to how the weights might be determined and this will be one of the quests in this article to explore different methods. The simplest, which assumes equal weights, is where the layers are used to filter out areas that are most suitable for developing and using a process akin to ‘sieving’. Where the scale chosen is from ‘0’ to ‘1’ with ‘0’ the land deemed most unsuitable, the layers can be added up to form a suitability surface, which is simply a composite of each layer. In fact, in methods such as these, the combinations of layers can be strengthened, reinforced or even weakened as the process of synthesis or combination takes place.

## 3. Synthesizing and weighting map overlays

These kinds of design method involve defining a series of map layers that can be associated with individual factors or features that affect the location problem. These factors must be comparable, and this usually means that they are commensurate in the sense of being capable of being combined in some way. This often means that they are measured and mapped with respect to every location comprising the landscape in question, and in this sense, the suitability associated with the same location in each layer is directly comparable. Thus, the suitability scores for each layer can be summed and averaged. Nesting this process in a wider context that is supported by GIS and its generalization to geodesign [11] relates this class of methods to factors that cannot be presented using map overlays although the examples we will present here are all directly reducible to spatial-locational layers which are comparable.

At the onset, we need to be clear about what constitutes the factors that we represent as map layers. The individual designer following the examples of Keeble [7] and McHarg [9] defines land suitability in terms of different factors such as physiography, accessibility, demography and so on that imply different suitabilities for development. As already noted, these we will refer to as *factors* (or features). We can also twist the problem slightly and consider the factors as being specific to individual *actors* or even *agents* where we argue that the combination of factors is akin to a process of resolving conflicts about land suitability, which is a process of averaging, pooling and/or transforming the opinions of different agents. So, for example, we might consider one designer as being concerned with, say, road accessibility, and another designer with landscape topography, where the process of resolving the difference between the two factors as one where the actors come to some consensus (or not, as the case may be).

To illustrate this process, let us define a factor as a land suitability map $A_{xy}^{k}$, k=1,2,…,n, where the factor is k and the locations defining the map of the factor are coordinates x,y. The map itself defines each location with the factor’s suitability score. Now the simplest process is to sum the suitability maps to produce a simple average as ${\overset{-}{A}}_{xy}=\sum_{k}A_{xy}^{k}/n$. Each map can be normalized to sum to 1, say, and this enables direct comparisons to be made of the factors and the ultimate land suitability ${\overset{-}{A}}_{xy}$. However, it is more usual to define a weighting for each factor as $w^{k}$ and then determine the overall suitability as a weighted average ${\hat{A}}_{xy}$, which is:


(3.1) $${\hat{A}}_{xy}=\sum_{k}w^{k}A_{xy}^{k}\;where\;\sum_{k}w^{k}=1\;.$$

There are a number of different ways of scaling land suitability scores before they are weighted and averaged or through the weights themselves. Each map might be scaled to a standard range, or the weights themselves might determine the range. In fact, both can be done with the maps being normalized and the weights being imposed when these are averaged.

However, a deeper analysis might be attempted in which the relationships between the factors are explored on the assumption that the relative similarity between the factors could be measured and then used to determine the order in which the factors might be combined. For example, if we had 10 factors and five were very close in terms of their spatial suitability, then we might merge these five first and then, dependent on how close the merged average so far is to the remaining factors, use these similarities to conclude the averaging in the remaining order. This method was first introduced by Alexander [12], who suggested that any spatial design problem should first be broken down into its component or constituent parts, in this context its relevant factors, which might be the different map layers. Alexander argued that the designer should consider the division of the problem into its parts as being a division of the system into subsystems or problem into subproblems. The first stage should be this decomposition into parts from the top-down and then the second, the assembly or synthesis of the parts back into subsystems that reflected the resolution of conflict between the components. This process of synthesis was to build a new system from an analysis of how strong the subsystems were in helping to resolve the problem. A worked example to the method for locating the route of a highway, similar to McHarg’s method, was first presented by Alexander & Manheim [13].

**Figure 3.**
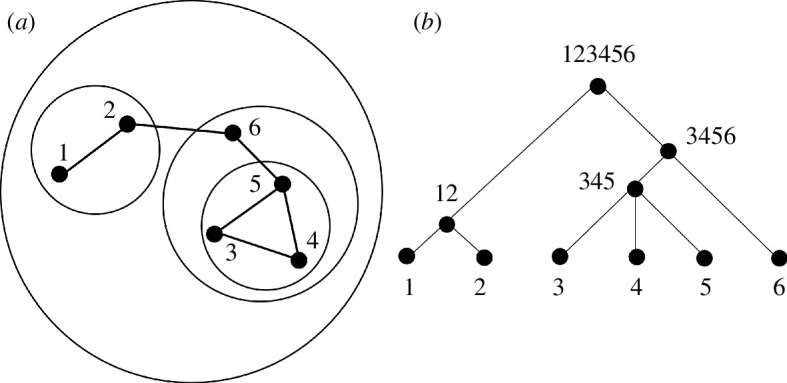
(*a*) A hypothetical example of clustered design factors. (*b*) A hierarchical ordering based on combining the factors according to the subsystem structure in (*a*).

Imagine the design problem could be defined by six elemental components and imagine that these components relate to one another in the manner illustrated by the system–subsystem diagram in figure 3*a*. Factor 1 is closely related to factor 2 and we might consider these to be two different kinds of accessibility where the highest location of accessibility is the most suitable for location. Factors 3, 4 and 5 are also closely related and these might be based on topographic slopes, land liable to flooding and risk of subsidence, again where their higher suitability is where these factors are not prevalent. Factor 6 is somewhere between the first and second clusters and might be a factor inhibiting development by a green belt, say. Now when we graph these relationships, we see a clear hierarchical structure that takes the topological graph in figure 3*a* and orders it as a hierarchy in figure 3*b*. Alexander [12] suggests that this hierarchy gives us an order to proceed. Note we have not developed a quantitative method for putting these factors together and we can assume that it is the intuition of the designer who defines this structure. In fact, we will illustrate a quantitative method with some real map overlays in the next section but for the moment, let us assume that we have a land suitability map for each of these factors: that is, we have $A_{xy}^{1},\;A_{xy}^{2},\;A_{xy}^{3},\;A_{xy}^{4},\;A_{xy}^{5}$ and$A_{xy}^{6}$ and the order given by the hierarchy in figure 3*b* shows how we combine each of these maps, noting that the process of combination is akin to finding a rational compromise—an average—between the factors as grouped into their subsystems.

**Figure 4.**
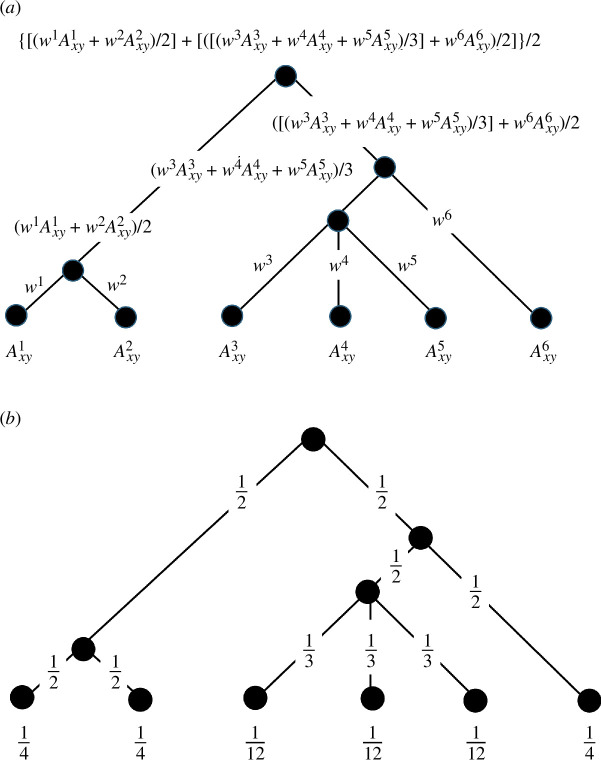
(*a*) The explicit weighting structure for the hierarchical composition and (*b*) the implicit weighting structure for the hierarchical decomposition.

The compromise at each stage represents a partial solution to the subproblem defined by the nested cluster so far and eventually the problem is solved as the designer’s process of compromise moves to the top of the tree. Now we first average factors 1 and 2, and then 3, 4 and 5 to generate average factors for the two subsystems. Then we average factor 6 against the subsystem of factors 3, 4 and 5, and then we combine this set with the average of factors 1 and 2 and this then gives us the final land suitability. We show this procedure in figure 4*a* where we note that at the base of the hierarchy, we define each link with a base weight $w^{k}.$ Note that these weights can be determined to reflect the strengths of relationship but for the moment we simply list the order in which the maps $A_{xy}^{k}$ are combined. The order of combination is as follows.

Factors 1 and 2 are merged from the first subset to form ${(w}^{1}A_{xy}^{1}+{w^{2}A}_{xy}^{2})/2$ while the second subsystem is a simple average of the third, fourth and fifth factors, which give $(w^{3}A_{xy}^{3}+w^{4}A_{xy}^{4}+w^{5}A_{xy}^{5})/3$. We then add factor 6 into this second subsystem to generate the new average $\left(\left[\left(w^{3}A_{xy}^{3}+w^{4}A_{xy}^{4}+w^{5}A_{xy}^{5}\right)/3\right]+w^{6}A_{xy}^{6}\right)/2.$ This takes us to the top of the tree where we merge the original 2-factor subsystem with the now 4-factor subsystem to create the final average: the compromise or the consensus is thus defined as $\{[(w^{1}A_{xy}^{1}+w^{2}A_{xy}^{2})/2]+\;[([(w^{3}A_{xy}^{3}+w^{4}A_{xy}^{4}+w^{5}A_{xy}^{5})/3]+w^{6}A_{xy}^{6})/2]\}/2.$ This is the final average, the consensus suitability, or the compromise associated with the structure of the problem defined in figure 4*a*.

We can now specify in detail the weighting structure in this kind of hierarchal design problem because we have two separate systems: first the simple averaging that could be weighted as we combine the branches and the weights for each of the bottom branches $w^{k}$ that drive the averaging as we proceed up the tree. Now if we were to set these weights to the number of branches being combined where $w^{1}=1/2$ and $w^{2}=1/2$, $w^{3}=1/3$, $w^{4}=1/3$ and $w^{5}=1/3$ and then $w^{6}=1/2$, then we can pass these up the tree defining new weights. This process is clearer if we descend the tree where it is clear that the simple averaging is preserved according to the number of branches at each level of the hierarchy. We show this in figure 4*b*.

Alexander’s [12] process of hierarchical design is essentially one in which problems are first defined from the top-down and then solved from the bottom-up. This is a process akin to analysis followed by synthesis or decomposition followed by composition. The general idea is that design first articulates the problem as one where a series of modules or elements—factors, features, layers, here—are first identified and the conflicts and/or correspondences between them measured. The method of weighting we have noted for such problems is essentially structural depending on the way the system is decomposed (and the composed) through the correlations or similarities between the layers. In fact, there are other methods for deriving the hierarchical weights with one of the most widely used due to Saaty [14] whose analytic hierarchy method provides a potentially consistent way of measuring the relationships between each layer or actor or factor. This is through a subjective but logical process of pairwise comparisons from which relative weights associated with each factor can be extracted. The method can be very effective although it is never completely consistent. It can however be nested in a predefined hierarchy with the overall procedure being used over and over again in an effort to improve its consistency. Although developed 40 years ago, it has not so far as we are aware been used to think about design problems in the way we have articulated them here, although there is still considerable potential for adapting them to different hierarchical problem-solving structures. One of the authors has worked with these methods in the context of extracting the relative weights of opinions on problems where stakeholders need to resolve conflicts, and these appear promising for design [15].

## 4. Exploring design problems through dissimilarity clustering

To demonstrate how we might find the best location for large-scale urban development, we will assemble a series of suitability maps that we can relate to each other, and which can be used to measure the strength of their relationships, from which we can infer an order in which the maps might be merged. We will define 19 such layers that can all be represented in the form of gridded data where each grid cell defining a location in the layer provides an index of land suitability. The measures can vary across a wide range of scores with their absolute values being compared between layers. In fact, here we will normalize each layer taking the largest and smallest values in each layer and then scaling them to vary between a maximum suitability of 100 and a minimum of 0. Even though this makes the assumption that each measure is directly comparable, we are able to specify differential weights between surfaces that give layers differential weighting, as we noted in the example that we introduced in the previous section.

**Figure 5.**
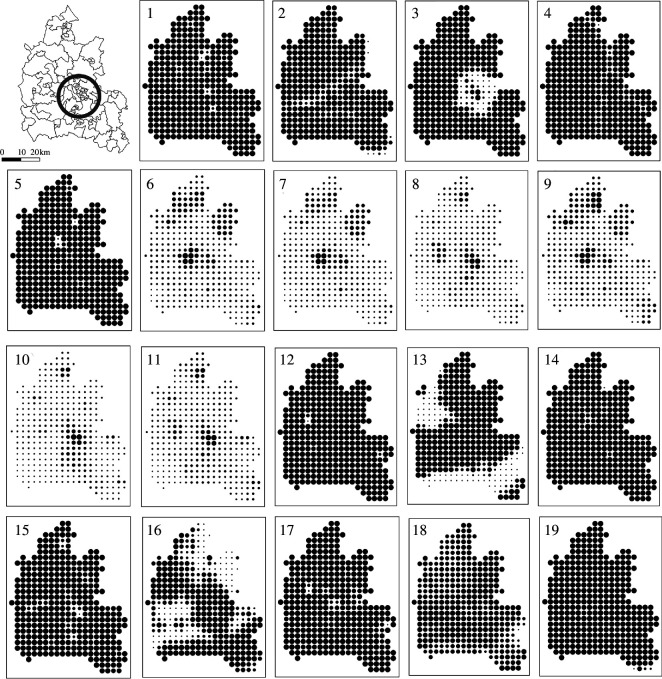
The land suitability layers for Oxfordshire. Note that the open circle in the top left corner of the grid of layers is Oxford.

Our example involves assessing the most suitable land for urban and housing development in the Oxford region (the County), which is almost 60 miles from central London and has a population of nearly three-quarters of a million. The area of the county is some 1000 square miles. The data we have assembled are from a variety of sources, geodemographic, physical and transportation-related, and we have divided the region (the County of Oxfordshire) into a regular 1 km^2^ grid of 353 zones where we measure the average suitability for development in each grid square according to each of the 19 layers of land suitability. We list these layers in table 1, where we also show the ultimate weightings used for various methods of averaging that we will develop in the rest of this article. The different layers are shown in figure 5 where the county of Oxfordshire, our case study for which we have the different measures of land suitability, is shown in terms of its division into 86 middle-layer super-output areas that define the locations for the geodemographic data. This is the first map in the top left of this figure.

**Table 1.** List of factors and differential weighting schemes.

layer	factor/actor/feature	equal weights	hierarchical weighting	opinion pooling [12]	opinion pooling [13]
1	conservation area	5.26	6.25	4.02	4.88
2	flood risk area	5.26	12.50	4.28	5.05
3	greenbelt	5.26	1.56	4.55	4.77
4	highway coverage	5.26	0.78	3.97	4.82
5	historical conservation	5.26	6.25	2.70	5.27
6	housing accessibility by bus	5.26	12.50	6.62	6.01
7	housing accessibility by rail	5.26	3.13	6.83	5.99
8	housing accessibility by road	5.26	1.56	7.77	5.69
9	job accessibility by bus	5.26	1.56	6.81	5.97
10	job accessibility by rail	5.26	0.78	7.81	5.63
11	job accessibility by road	5.26	3.13	7.84	5.71
12	national nature reserves	5.26	6.25	3.07	5.39
13	outstanding natural beauty	5.26	6.25	5.81	5.24
14	parks and gardens	5.26	12.50	3.11	5.28
15	population density	5.26	1.56	6.73	4.18
16	proximity to green space	5.26	3.13	5.47	4.40
17	special scientific interest	5.26	1.56	5.66	5.10
18	slope	5.26	6.25	4.06	5.39
19	surface water	5.26	12.50	2.90	5.24

The 19 layers defined in table 1 and mapped in figure 5 tend to cluster into three groups, typical of the standard set of indices that determine the suitability of different locations for urban development [9]. The list of factors in table 1 cover physical factors such as flood risk, slope and surface water, which in turn are complemented by a second set based on green space such as conservation, green belt, nature reserves, parks and gardens, proximity to green space and sites of special scientific interest. This second set are physical in nature but are institutionalized into areas that in various ways are protected from development. The remaining and largest clusters are factors relating to urban development, in particular highways, accessibility to different locations associated with different modes of transport and population density. Some of these factors cover much larger areas of the region than others, so areas of outstanding natural beauty and green belts are extensive.

The other key features that can easily be seen from the layers themselves are factors that have highly localized suitabilities or otherwise for development while others cover the entire region. In short, some layers have low and high suitability in a few very specific locations while other highs and lows cover much more area of the whole region. Note that the circular locations defined for each map on the grid vary in size in proportion to their intensity which is their suitability for development (with the biggest dots the most suitable).

**Figure 6.**
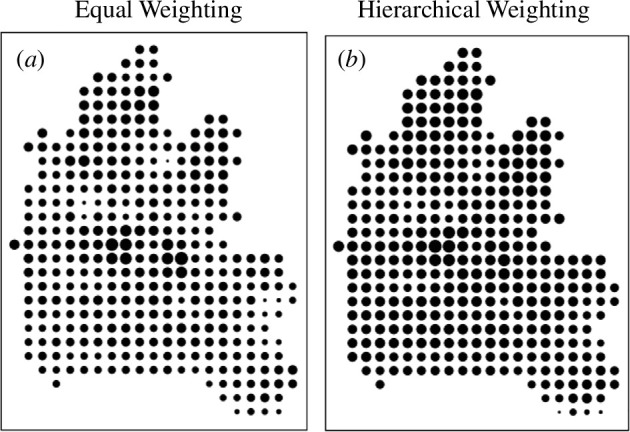
(*a*) Equal and (*b*) hierarchical weights.

The baseline solution to this problem is based on giving the same weight to each of the layers, using these weights to scale them and then summing them according to equation (3.1), which we repeat with explicit equal weights for each layer, k as $w^{k}=1/n$, ∀k, as ${\overset{-}{A}}_{xy}=\sum_{k}{\frac{1}{n}A}_{xy}^{k}$. This is the most obvious solution from simply adding the map layers together and noting that each layer is commensurate with every other due to the fact that each individual suitability is scaled over the same range $0\leq A_{xy}^{k}\leq 100$. We show this default solution in figure 6*a*, where if you examine the 19 layers in figure 5, it is very clear that the areas around Oxford itself are the most suitable locations for new development and these are areas of highest accessibility. In figure 6*a*, the conservation areas are reinforced as the least suitable areas for development, as expected. It is worth noting that this problem is rather highly structured in terms of the differences between the map layers although what this approach emphasizes is that the designer should use these solutions to explore the detailed issues of land suitability which the composite layer identifies. Here, we are not able to explore the problem in its fullest way for this would involve us looking at every possible combination of layers, which would represent a sensitivity analysis of the problem. What we will do however is use two additional weighting methods, the first which explores the structure of relationships between the individual layers utilizing the hierarchical methods introduced in the previous section.

**Figure 7.**
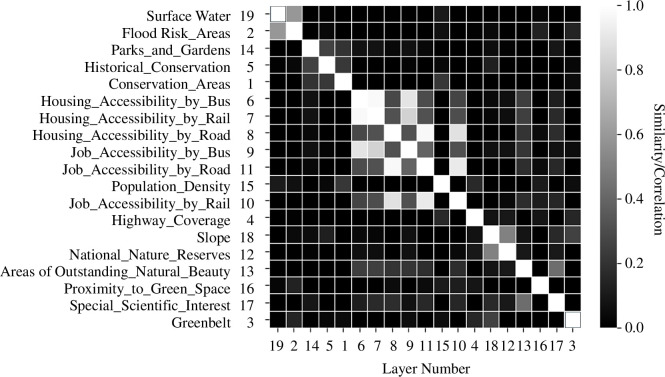
The reordered matrix of relationships based on spatial correlations.

To explore the structure of the problem, we need to examine the relationships between all 19 layers and extract any patterns that define the set of relationships. We will use the method first introduced by Alexander [12] where we measure the relationships between the layers and then decompose the problem into subsets which reflect the density of these relationships by identifying a hierarchy of clusters. There are many ways of doing this, but the key is to identify the nature of the relationships between any pair of factors. In this context, the relationships that we seek are similarities between the factors and we consider that their spatial similarities between a layer and every other constitutes the basis for a decomposition and extraction of a hierarchy. We thus measure the set of relationships between 19 layers using spatial similarity measures and, in this case, we measure the standard correlation between each layer. Each layer has 353 values of similarity (across the grid), and it is these that form the basis for the cluster analysis. We first compute the matrix of correlation coefficients, but this has to be reordered so that we can extract the density of relationships with the densest forming clusters that can be arranged in hierarchical fashion. The matrix, as shown in figure 7, has been reordered using a standard method of clustering [16]. This is referred to as single linkage analysis and consists of first identifying the two layers that are most similar, then merging these variables and recomputing the correlations between the first group and the other layers. With this new matrix, which now consists of 17 layers and two in the first cluster, the next highest similarity is identified, and a new cluster is formed in a similar way. In this way, the layers are slowly merged and progressively reduced usually one at a time until all the layers are combined. The tree or dendrogram that emerges is then used to compute the weighting of its branches, which determines the order in which the layers are combined, as we show in figure 4*a*,*b*.

**Figure 8.**
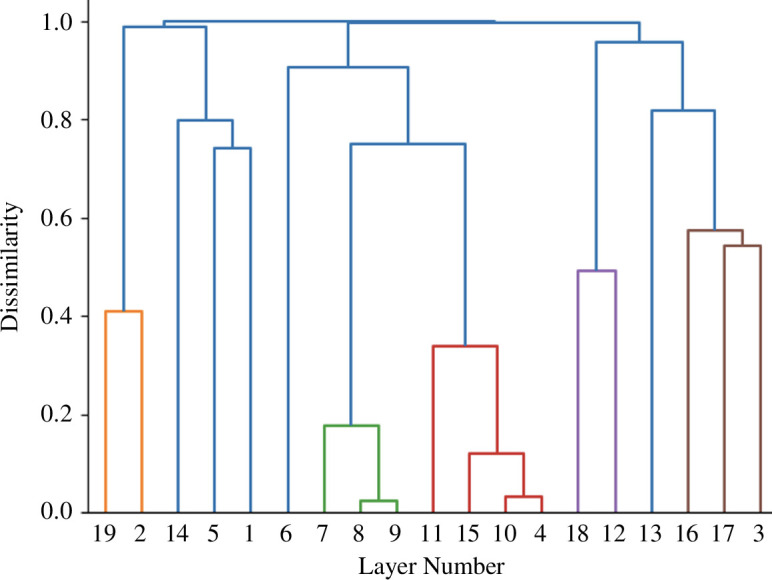
Translating the cluster matrix into the hierarchical composition.

The correlation matrix is reordered to ensure that the highest and lowest correlation values are clustered as closely as possible along the main diagonal, but to display the hierarchical order it is necessary to form a measure of dissimilarity rather than similarity. The easiest way to do this is to form the measure δ(k1,k2)=1-ρ(k1,k2), where ρ(k1,k2) is the correlation coefficient between layers k1 and k2. The tree formed from this process in figure 8 where basic clusters combining two or more layers are merged and reveal a very clear and intuitively satisfactory analytical result. For example, the basic clusters are 19 and 2, 5 and 1, 8 and 9, 10 and 4, 18 and 12 and 17 and 3, and these make good empirical sense when the types of layers are considered. In fact, the main clusters are identified where the levels of dissimilarity are less than 0.6 (or similarity greater than 0.4). The blue lines in figure 8 are the most dissimilar clusters while the coloured branches—orange, green, red, purple and brown—define layers associated with water, housing accessibility, density and job accessibility, slope and green space.

We have not attempted to illustrate this graph as being subdivided into problem and subproblem clusters largely because these are fairly clear from the tree and the matrix themselves. The order that figure 8 suggests defines a sequence of probabilities along the branches of the tree from the top-down that defines the set of weights for the layers that we list in table 1. Of course, the correlation with the equal weighting scheme whose output we illustrated in figure 6*a* is effectively zero, but if you compare the composite suitability indices in terms of the spatial correlation between figure 6*a*,*b* this is quite high at 0.797. This is an important point in that although the weights from the hierarchical averaging are quite different from the equal weighting, the similarities/correlations between the maps are quite high and this is likely to be a consequence of the fact that the accessibility maps (layers 4, 6–11) reinforce one another, then the physical maps reinforce each other in almost an opposite manner and the other layers are quite uniform and do not contribute much to the final variation. This suggests that in future developments of these sorts of method, we need to identify weights for different locations (grid squares) rather than for entire layers. There are implications here for adapting Saaty’s [14] method of pairwise comparisons to enrich this process.

## 5. Transforming design problems into networks for opinion pooling

So far, we have been at pains to emphasize the way we have conceived of the problem of land development as being based on the idea that designers hold individual opinions about potential solutions to the problem that they, individually, need to reconcile in their search for an optimum. In fact, in the sorts of problems we have sketched, it is likely that more than one individual would be involved in mobilizing the data and arguing out the solution to the problem in terms of the way they might reach a consensus over the intrinsic differences or conflicts implied by each individual factor or feature. What we will do now is generalize the problem to one where each individual factor $A_{xy}^{k}$ is associated with an actor or designer k where the process of averaging or reaching a compromise involves each designer swapping their own factor/layer with those designers holding other factors/layers $A_{xy}^{\ell },\ell \neq k$. To show how this might work, we envisage a strictly regimented process where at any time period, each actor swaps his or her factor with those actors to whom she is connected, thus producing a new factor average. The actor’s new factor would thus be a weighted average of those factors to which she is linked, and the process would continue in the same way with the actor’s average factor getting closer and closer to the average of all those actors to which she is linked. If every actor is connected to every other actor, then the average of factors for all actors would be a simple average generated in one pass of the process. It is much more likely however that although the actors are connected, they are not completely so, and thus the process would converge on a weighted average that would be a more complex reflection of the network links among all the actors.

**Figure 9.**
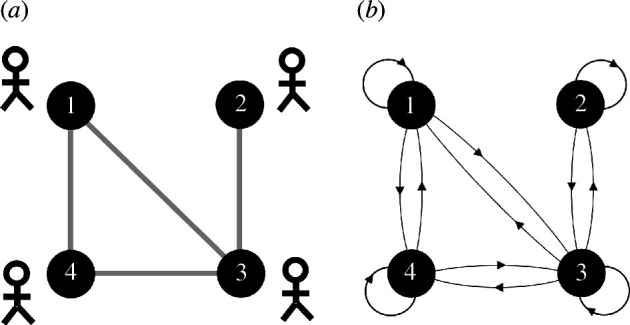
(*a*) Actor-factor network. (*b*) Transmission: opinion-pooling.

We show a very simple network linking four actors (and their factors) in figure 9*a*,*b*. The network in figure 9*a* is strongly connected meaning any actor can transmit messages to the other three, either directly or indirectly. We assume the links are symmetric and in figure 9*b* we show the actual links for message transmission, noting that at each stage of the averaging, the actors pass their land suitability layer to those to whom they are linked, including themselves, which is the self-link. You can easily guess that the initial differences are ironed out and eventually a weighted average occurs that is the same for each actor. Real networks are likely to be much bigger than this ‘toy’ network, and in these cases, there could be many different kinds of network links where the actors generate different weighted structures. These can range from a situation where everyone is connected to everyone else and a simple average emerges, through to situations where the network is disconnected into subnetworks and no overall average occurs. Situations where one or a group of actors transmit their factors but are not influenced by other actors, can also occur, and in this latter case, the actors transmit their messages continually and their opinions thus dominate the eventual outcome. We illustrate this in a little more detail elsewhere [17].

**Figure 10.**
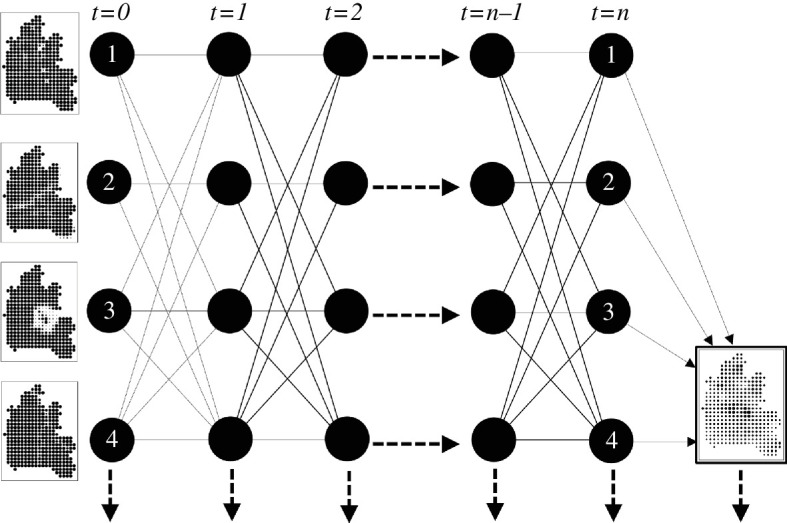
Averaging and opinion-pooling through forward propagation.

We show the process of transmission in figure 10, which is reminiscent of a neural net with simple forward propagation of the weighted links and layers. It is quite straightforward to formalize this process, but before we do so, it is important to consider how the network links between actors can be defined. The most obvious way is by direct observations of who connects or influences who but behind these connections lie somewhat deeper reasons for connection. In this case, we have already produced a matrix of connections between factors based on computing the spatial correlations between factors, and we could use the strength of these links to form the sizes of the channels linking actors together. In this case, every actor would be related to every other actor, but the links would be weighted and normalized to reflect the strength of the correlations. For example, if actor k were linked to all other actors ℓ according to the strength of their correlation ρ(k,ℓ), then we could normalize these correlations into weights, which for each actor could sum to 1. In short, for actor k, her weights to all other actors ℓ sum to 1, that is $\sum_{\ell }w_{k\ell }=1.$ The network is thus composed of a set or normalized weights, which in matrix form is $W=\left[w_{k\ell }\right]$.

We can now write the averaging process as follows. We begin with the usual set of land suitability layers $A_{xy}^{\ell }\left(t=1\right)$ where t is the temporal index, and we form the average for each layer at the next time interval $A_{xy}^{\ell }\left(t=2\right)$ by applying the weights matrix W. The generic equation for each time interval is thus

(5.1) $$A_{xy}^{k}\left(t+1\right)=\sum_{l}w_{k\ell }A_{xy}^{\ell }\left(t\right),$$

which in matrix form can be written as:


(5.2) $$A_{xy}\left(t+1\right)=WA_{xy}\left(t\right).$$

Recurrence on equation (5.2) leads to


(5.3) $$A_{xy}(t+n)=W^{n}A_{xy}(1)$$

and from the properties of W, which is a stochastic Markov matrix, the limit of the recurrence on equation (5.3) can be written as:


(5.4) $${\overset{-}{A}}_{xy}=ZA_{xy}\left(1\right).$$

$Z=W^{n},n\to \infty$ is the limit matrix where each row is identical and ${\overset{-}{A}}_{xy}$ is the averaged layer or factor that each actor generates through the process. This is no more than a formal representation of the averaging process where each actor averages his or her factor with respect to all the other actors to whom she is linked at each iteration.

We can illustrate this process using the 19 layers of land suitability that we generated for Oxfordshire. Our first network is based on the correlations between all 19 layers where we form the matrix W by taking the absolute values of each individual correlation $\left|W\right|$ =$\left|w_{k\ell }\right|$. In essence what this means is that one layer must be compromised or averaged with another layer if their spatial correlation is high or low, thresholds which can be fixed at a given value such as 0.5 or −0.5 but which can also depend on the analyst’s subjective interpretation of the problem. Factors that conflict with one another with negative correlations must be merged at the same time as those factors that have high correlations. In our second variant, we renormalize the correlation matrix so that the lowest correlation is set to 0 and the highest to 1 and this indicates that the weights relating any two factors are proportionate to the normalized correlations $w_{k\ell }=\left[\rho \left(k,\ell \right)-\min\rho \right]/\left[\max\rho -\min\rho \right]$. This weighting is based on averaging the factors according to their similarity whereas the first method assumes that those with greatest and least similarity are considered equivalent.

**Figure 11.**
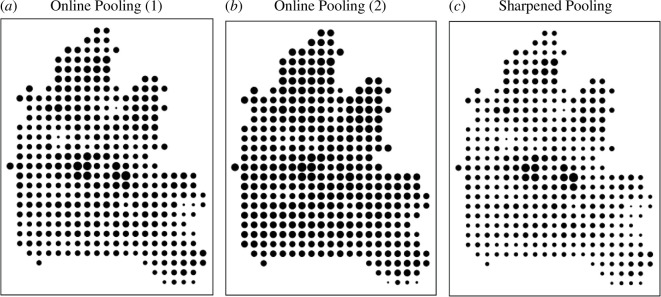
Solutions associated with opinion pooling (*a*) based on $\left|W\right|$, (*b*) based on W, (*c*) based on W and on sharpening ${\left(A_{xy}^{\ell }\right)}^{3}$.

The two averaged layers for these sets of weights that are referred to as opinion pooling in [12] and [13] are shown in figure 11. In fact, they are very similar, and this is probably due to the fact that the spatial variations in the original set of 19 layers show considerable joint correlation with respect to their suitabilities but also that the process of merging/averaging reduces the distance between the various indices. In fact, the overall correlation between the maps in figure 11*a*,*b* is 0.97. To an extent, the impact of this averaging could be increased quite dramatically by sharpening the range of land suitability values and renormalizing. For example, if the intensity of the averaged land suitability were increased by setting the final average as ${\left(A_{xy}^{\ell }\right)}^{\beta }$ where we set β>1 (or even sharpen each measure before the initial averaging takes place), then this would increase the discriminatory power of the method to identify the most suitable areas. This does not change the method as such, but it does communicate the solutions more clearly and in figure 11*c* we show figure 11*b* substantially sharpened with β set at 3.

The model we have described has its origins in a ‘formal theory of social power’ first articulated by French [18] and set within a process in which conflict between different views held by different actors is resolved using linear averaging. The process was formalized as a simple Markov chain by Harary [19] and was then elaborated in different ways but within the context of the Markov model by DeGroot [20], Kelly [21] and others. It was first applied to resolving spatial conflicts by one of the authors [2,22], and it has subsequently been used in diverse contexts involving models of opinion dynamics, specifically opinion pooling, where its equilibrium properties have been explored in detail (see [23]).

As the model has a simple linear form, it can be easily expanded to include exogenous variables at each stage of consensus formation. For example, at each iteration, a fixed external factor, which we define for each actor as $A_{xy}$ can be added, which might represent a set of suitability indices that never change but are relevant to the ultimate solution. The model in equation (5.2) can thus be written as $A_{xy}\left(t+1\right)=WA_{xy}\left(t\right)+A_{xy}$. A variant of this has been explored for a process of social influence by Friedkin & Johnsen [24] and then generalized to a system where the network of social influence is weighted against the external factors. This yields the equation $A_{xy}\left(t+1\right)=\Lambda WA_{xy}\left(t\right)+{(I-\Lambda )A}_{xy}$, where Λ is a diagonal matrix of weights between 0 and 1 [25]. There are many other variants of this model but in this context, more important developments relate to how the formal model might be applied empirically to networks of stakeholders involved in producing plans or designs, in this case for various kinds of urban development. We will conclude by presenting some of the main extensions to these ideas.

## 6. Future developments of the participatory model

The model we have introduced is highly stylized in that the actors (or factors) are uniform in their behaviour and the process of averaging or opinion pooling is strictly organized to take place in each time period or iteration. We could introduce all kinds of constraints on message passing using the channels of the social network, but here we simply assume that there are no obstacles to swapping opinions. If we were to tune this model to an empirical situation, we would need considerable data on the behaviour of actors, on any changes in the network as the process continues, and any changes in the way actors make a compromise or move towards a consensus or not in each pass through the network. We have not explored the possibilities of doing this with the model, but we do have some experience of working in a semi-formal context with stakeholders—designers—who are engaged in producing design in teams that are close to groups of actors interacting on a semi-normalized network. Steinitz [26] in his applications of geodesign to large-scale urban development at the regional scale illustrates how one might progress towards empirical applications of this kind of model with future work focused on improving towards the goal of modelling the geodesign process as one of opinion pooling. In such extensions, we would introduce a variety of structured processes involving the way groups of actors in the geodesign process would interact with further empirical development of the opinion network pooling models introduced here.

We have structured the model so that it will always reach equilibrium, but many readers will immediately raise the prospect that most design problems never reach such a condition, notwithstanding that many such problems are solved with arbitrary decisions made about what the equilibrium should be. We have not explored different kinds of equilibrium, disequilibrium or any variant such as far-from-equilibrium that characterizes such location problems although we have provided hints at how such models can be so generalized [17]. In fact, in all planning problems, there is a distinction between the nature of an optimal locational solution that is the equilibrium produced by the model and the stability of the network which is an equilibrium outcome of the process of averaging. Thus, both the process and the product that is the spatial outcome of the process are in equilibrium. Any element of this nexus of product and process can be varied to change the nature of the design solution. Future applications of these ideas will engage with such extensions.

When we consider tuning this model to an empirical application, it is very likely that the number of stakeholders or designers would be much greater than the example shown here with hundreds rather than tens of stakeholders, hence hundreds of factors. The network that links themselves would then scale geometrically in the order of *n*^2^. There are no problems of computation for these models run very quickly but the data pertaining to what the stakeholders consider in terms of land suitability for development are hard to identify and measure and even getting access to this expertise, representing it and then modelling it, is difficult. There are very few examples of moving and scaling these processes of design to formal models although a start has been made. This is another example in the field of city planning and design where there are many oblique perspectives on what constitutes optimality in design in contrast to what we consider to be optimal designs. An inquiry into different modes of optimality in design is urgently needed with respect to the wider context introduced here.

## Data Availability

Software for the Oxfordshire case study of the Land Development Model (LDM) is available for download [27].
